# Tribological performance of mechanical face seals for Martian applications

**DOI:** 10.1038/s41598-026-48495-2

**Published:** 2026-04-14

**Authors:** Péter Marcell Kiss, Gábor Kalácska, Róbert Zsolt Keresztes, Zoltán Károly

**Affiliations:** 1 Mechanical Engineering Doctoral Program, Magyar Agrár- és Élettudományi Egyetem (MATE), Páter K. u. 1., Gödöllő, H-2100 Hungary; 2 Institute of Technology, Szent István Campus, Magyar Agrár- és Élettudományi Egyetem (MATE), Páter K. u. 1., Gödöllő, H-2100 Hungary; 3 Institute of Materials and Environmental Chemistry, HUN-REN Research Centre for Natural Sciences, Magyar Tudósok krt. 2., Budapest, H-1117 Hungary

**Keywords:** Tribology, Mechanical seal, Martian simulant, Wear mechanisms, Engineering, Materials science

## Abstract

This study examines the tribological performance of dry-running mechanical face seals composed of a graphite rotating ring and a silicon carbide stationary ring under varying contact pressure conditions from 0.23 to 0.83 MPa), both in clean operation and in the presence of a Martian regolith simulant (MGS-1, PR < 80 μm). Three preload levels were applied: zero preload (SP0), nominal preload (SP1), and double preload (SP2), based on a calibrated spring characteristic and contact area analysis. Under clean conditions, increasing preload elevated frictional torque from ~ 0.017 Nm (SP0) to ~ 0.030 Nm (SP1) and ~ 0.070 Nm (SP2), indicating a direct correlation between contact pressure and interfacial shear. When MGS-1 regolith simulant was introduced, torque increased significantly in all configurations, reaching ~ 0.064 Nm (SP0MGS-1), ~ 0.082 Nm (SP1MGS-1), and ~ 0.095 Nm (SP2MGS-1). Real-time monitoring of torque and axial force enabled the calculation of dynamic friction forces and coefficients of friction, which revealed that contact pressure strongly influenced wear mechanisms: higher contact pressure enhanced interface conformity but accelerated abrasive degradation, while lower contact pressure reduced wear at the expense of sealing pressure. Scanning electron microscopy (SEM) confirmed the presence of abrasive grooves and embedded particles exclusively on the graphite ring, with no perceivable damage on the SiC counterface. The results demonstrate that nominal contact pressure (SP1) provides the most favorable balance between friction stability, wear resistance, and sealing reliability, offering valuable insights for the design of mechanical seals in particulate-laden environments such as Martian surface operations.

**Supplementary Information:**

The online version contains supplementary material available at 10.1038/s41598-026-48495-2.

## Introduction

Mechanical face seals are critical components used to prevent fluid or gas leakage between rotating and stationary parts in a wide range of engineering systems. These seals are commonly found in pumps, compressors, and other rotating equipment where reliability and performance are essential. Their basic design consists of two flat, smooth sealing surfaces one rotating and one stationary that are pressed together by a combination of spring force and fluid pressure to form a tight, leak-proof interface. The effectiveness of a mechanical seal depends heavily on material selection, surface finish, load distribution, and environmental conditions such as temperature, pressure, and the presence of abrasive particles. In demanding environments like those found in aerospace or planetary exploration, the design requirements for these seals become even more stringent^1–5^.

Mars regolith, a fine and abrasive soil, poses a significant threat to sealing integrity due to its potential to cause wear and infiltration. The MGS-1 (Mars Global Simulant) is a scientifically developed regolith simulant designed to mimic the physical and chemical properties of Martian soil. It is used in laboratory tests to evaluate the performance and durability of mechanical systems, including face seals, under Mars-like conditions^6,7^.

Mechanical face seals could play a crucial role in protecting electromechanical components of Mars rovers, such as actuators, motors, and joints, from environmental contamination. These components are vital for mobility, manipulation, and instrument operation, and their protection is essential for the overall functionality and longevity of the vehicle. Without effective sealing, fine Martian dust could penetrate internal assemblies, leading to increased friction, component failure, and mission risk^8,9^. Mars presents an especially hostile operating environment for mechanical systems^10^. The atmospheric pressure is less than 1% of Earth’s, resulting in reduced convective cooling and unique tribological behavior in seals. Wind-driven dust storms can last for weeks and carry sharp, electrostatically charged particles that further challenge seal performance. By testing mechanical seals with MGS-1, engineers can better design dust-resistant, temperature-tolerant, and pressure-resilient seals. These advancements are key to ensuring the operational reliability of future robotic missions and potentially human exploration systems on Mars.

Silicon carbide offers excellent hardness, chemical inertness, and dimensional stability under elevated temperatures, making it highly suitable for use as the stationary component in mechanical seals^11,12^. Its low thermal expansion and high thermal conductivity further contribute to reliable operation under fluctuating thermal and mechanical loads. The carbon-based sealing ring provides complementary tribological properties. Its inherent self-lubricating behavior, resulting from the layered structure of graphite, significantly reduces friction and wear during dry or marginally lubricated operation. This self-lubrication effect becomes particularly relevant under start-stop conditions or during lubrication film breakdown, enhancing the durability of the seal and minimizing energy losses^13^. The combination of a hard, chemically stable stationary ring and a self-lubricating rotating counterpart ensures a robust sealing interface capable of withstanding challenging operational environments. This material pairing thus serves as a representative model for evaluating tribological behavior and wear mechanisms in the presence of a regolith simulant under Mars-like conditions.

The tribological characteristics of silicon carbide and graphite friction pairs have been extensively studied under terrestrial conditions, where they are valued for their excellent self-lubricating properties, high thermal conductivity, and chemical resistance^14,15^. While the lubricating mechanism of graphite is well-understood^16^, recent research has focused on the abrasive nature of Martian regolith, characterizing its mineralogical composition and its potential to cause severe surface degradation in space exploration hardware^17^. However, the coupled effect of these factors is a less explored area, as the systematic interaction between SiC-graphite seal pairs and Martian simulant under varying mechanical loads has not been fully investigated. In this context, the environmental sensitivity of graphite’s lubricity is a critical factor. It is stablished that graphite requires the adsorption of gas molecules, typically water vapor, oxygen, or CO_2_ to weaken the interlamellar van der Waals forces and facilitate low-friction sliding^16,18^. In high-vacuum environments, the absence of these contaminants leads to “dusting”, a phenomenon characterized by rapid wear and a sharp increase in friction^16^. However, the Martian surface is not a total vacuum; its low-pressure atmosphere is predominantly composed of CO_2_. Foundational studies by Savage^16^ and Lancaster^18^ have demonstrated that while the lubricating efficiency is lower than at terrestrial 1-bar pressure, the partial pressure of Martian CO_2_ is sufficient to provide the molecular adsorption necessary to prevent catastrophic dusting^18^. Thus, while the Martian atmosphere is less favorable than Earth’s, it represents a “bridge” environment that allows graphite to remain a viable solid lubricant, provided the additional abrasive impact of the regolith is accounted for.

In our research the aim was to assess the wear and performance behavior of the SiC - graphite seal pair under simulated Martian simulant exposure. The tribological interactions between the mechanical seal surfaces and fine abrasive particles such as those in MGS-1 are of critical importance for future planetary exploration equipment^19,20^.

## Materials and methods

The mechanical seal pair composed of a silicon carbide (RBSC) stationary ring and a carbon-based rotating ring (Calvo Sealing Ltd.) was selected for this study due to their widespread industrial use and well-documented performance characteristics^21^. These materials represent one of the most commonly applied combinations in dynamic sealing systems, particularly in environments requiring high wear resistance and thermal stability.

**Fig. 1:**
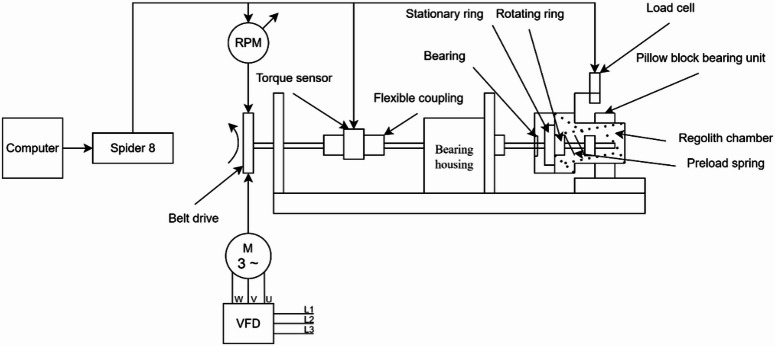
Schematic diagram of the test bench.

The experimental test bench is illustrated schematically in Fig. 1 and is designed to investigate the tribological behavior of mechanical face seals under controlled conditions. The system provides stable rotational speeds and precise torque monitoring throughout the long-term measurements. A detailed description of the drive system’s operating principle, the transmission configuration, and the sensor integration is provided in Appendix A.1.

**Fig. 2:**
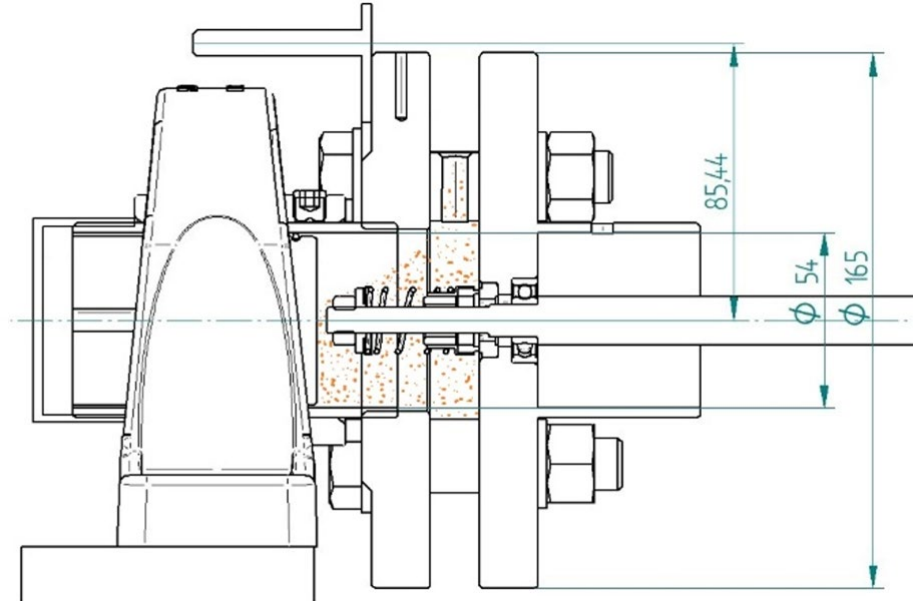
Cross-Sectional view of the mechanical seal test setup and simulant delivery into the chamber.

The sealing interface consists of a stationary Silicon Carbide (RBSiC) ring and a rotating resin-impregnated carbon-graphite ring. The stationary ring is mounted in a flange facing the dust chamber, while the rotating ring is fixed to the shaft. The positioning of the sealing rings within the dust chamber and the sequential process of filling it with regolith simulant are illustrated in Fig. 2.

Proper contact between the sealing faces is maintained by a precision-ground compression spring, which applies a constant axial preload. To ensure zero initial clearance, the assembly was performed manually, and the contact integrity was verified using a light-gap test before each run.

The experimental program was conducted in two phases. In the first phase, three reference test series (SP0, SP1, and SP2) were performed under clean, simulant-free conditions to establish baseline data. The SP0 configuration represents the baseline contact state. During assembly, the ring surfaces were brought into contact, but zero external spring pressure was applied. However, a measurable residual torque (0.017 Nm) was recorded even in this state. This indicates a baseline contact pressure arising from internal mechanical constraints, assembly hysteresis, and the self-weight of the components. Following the Amontons-Coulomb principle and considering the dry sliding of Silicon Carbide carbon-graphite interfaces^22^, this baseline serves as the initial reference point. The SP0, SP1, and SP2 settings thus represent baseline, nominal, and double preload conditions, respectively.

In the second phase, the tests were repeated with the dust chamber filled with 150 ml of MGS-1 regolith. To represent the most critical fine fraction, only particles sieved below 80 μm were used. Upon starting the rotation, the centrifugal effect ensures the uniform distribution of the simulant, leading to the continuous ingress of particles into the sealing interface. This dual-phase experimental approach enabled a systematic comparison between clean and regolith simulant-contaminated operating conditions, providing insights into the influence of fine Martian regolith simulant on the frictional behavior and potential wear mechanisms of mechanical seal systems.

All experiments were conducted at a constant shaft speed of 200 rpm under controlled laboratory conditions (22–24 °C, 40–50% RH). To evaluate the different wear mechanisms, a surface analysis was performed using scanning electron microscopy (SEM). The sealing components were documented in their as-manufactured state, as well as following the SP0, SP1, and SP2 test series conducted with MGS-1 regolith. This approach allowed for the identification of characteristic wear patterns, such as micro cutting or particle embedding, by comparing the pristine surfaces to the intermediate and final states of the sealing rings after exposure under different contact pressure to the regolith. Throughout each 24-hour run, the friction torque and axial force were recorded in real-time. The detailed specifications of the sealing geometry, the spring calibration values (Table 1), the calculation of friction torque and the simulant properties are provided in Appendix A.2, B.1, B.2 and C1.

The regolith simulant used in this study was Mars Global Simulant, a terrestrial analogue developed to reproduce the physical and mineralogical properties of Martian soil. The MGS-1 simulant is primarily composed of basaltic minerals, with the most abrasive components being olivine and pyroxene. The micro hardness of these mineral constituents ranges between 500 HV and 1000 HV. Comparing these values to the sealing materials, the SiC stationary ring (approx. 2200 HV) provides high resistance against abrasive wear, whereas the carbon-graphite rotating ring (approx. 80–100 HV) is susceptible to micro-cutting and particle embedding due to its lower hardness relative to the basaltic particles.

A total of 1500 g of the simulant was subjected to dry mechanical sieving in order to obtain discrete particle size fractions for experimental analysis. Significant characteristics of the selected regolith are provided in the Appendix C1, while the detailed characterization of the simulant is available in^17,21,23–25^.

## Results

Each experimental configuration (SP0, SP1, SP2, SP0MGS-1, SP01MGS-1, SP2MGS-1) was performed in triplicate. The data presented in the following sections represent a characteristic measurement series selected from these three independent repetitions. In all cases, the repeated tests under identical conditions consistently yielded the same trends. The reliability of the torque measurements was assessed by calculating the margin of error (at a 95% confidence level) for each data point based on 50 consecutive samples. The results demonstrate high measurement precision across all test groups. The average margin of error was found to be 2.44% for SP0, 1.06% for SP1, and 0.32% for SP2. The MGS-1 modified variants showed average error margins of 0.61% (SP0MGS-1), 0.82% (SP1MGS-1), and 0.65% (SP2MGS-1).

**Fig. 3:**
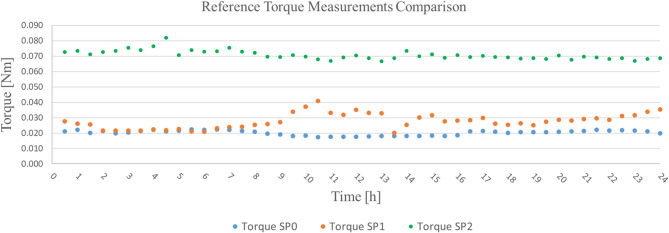
Measured torque over the 24-hour reference test for the SP0, SP1, SP2 configuration. Average margin of errors are 2.44% for SP0, 1.06% for SP1, and 0.32% for SP2.

Figure 3 illustrates the torque data obtained from online measurements for SP0, SP1 and SP2. It presents the time-dependent behavior of frictional torque measured in a series of dry-running mechanical face seal tests, performed with varying levels of contact pressures: baseline contact pressure (SP0), single contact pressure (SP1), and doubled contact pressure (SP2).

The points represent a characteristic measurement series, where repeated tests under identical conditions consistently yielded the same trends. In all configurations, the seal assembly consisted of a rotating graphite ring and a stationary silicon carbide counterface, and the system operated without any external lubrication under ambient conditions. The aim was to evaluate the influence of contact pressure on the tribological performance and torque characteristics of the seal pair during extended operation.

### SP0—baseline contact pressure

Figure 3 displays the torque profile for the SP0 configuration, in which baseline contact pressure was applied. During the initial phase of the test (0–3 h), the torque exhibited transient behavior with moderate fluctuations, indicative of a typical run-in process. This stage corresponds to the adaptation of the mating surfaces, where asperities are gradually flattened and a more stable contact interface is formed. The measured torque at SP0 (0.017 Nm) represents a system baseline, which includes both the residual contact pressure of the rings and the internal parasitic friction of the assembly. While this baseline torque yields a theoretical pressure of 0.23 MPa if attributed solely to the Silicon Carbide carbon-graphite interface (µ ≈ 0.15), its primary role in this study is to serve as a constant offset. Assuming a friction coefficient of µ ≈ 0.15 for dry Silicon Carbide carbon-graphite as per Miyoshi^26^ the effective baseline contact pressure (P_base_) was estimated using the following mechanical relationship:

$$\:{P}_{base}=\frac{{M}_{SP0}}{\mu\:\cdot\:{r}_{avg}\cdot\:{A}_{contact}}\:$$

where M_SP0_ ≈ 0.017 Nm is the measured residual torque, r_mean_ is the mean contact radius, and A_contact_ is the effective interfacial area. Given the ring dimensions (r_avg_ = 8.65 mm, A_contact_ = 57.07 mm^2^ ), this calculation yields a baseline pressure of approximately P_base_ = 0.23 MPa. While this value encompasses the entire assembly’s resistance, the linear increase in torque observed fromSP0 to SP1 and SP2 confirms that the system follows the Amontons-Coulomb friction model, where the additional torque is directly proportional to the incremental spring load.

This behavior is characteristic of dry-running graphite–silicon carbide pairs, where the inherent self-lubricating properties of graphite, coupled with the hardness and wear resistance of silicon carbide, yield favorable frictional conditions. At around 17 h, a noticeable increase in torque occurred, reaching a new steady-state level near 0.021 Nm. This transition likely signifies changes in the interfacial contact due to thermal effects, degradation of surface films, or gradual material wear. Despite the increase, the torque remained stable for the remainder of the test, confirming that the seal continued to operate reliably, albeit at a slightly elevated friction level. The presence of two distinct torque regimes initial low-friction followed by a higher-friction phase highlights the dynamic nature of surface evolution in dry contact.

### SP1—recommended contact pressure

Figure 3 shows the results for the SP1 configuration, where the contact pressure was set to the value recommended by the seal manufacturer. Accounting for the system baseline offset, this corresponds to a total effective contact pressure 0.528 MPa (Appendix Section B.2. Table 1). This adjustment had a clear impact on the tribological behavior. The run-in phase was shorter and more controlled compared to SP0, with the torque stabilizing around 0.020 Nm within the first 4 h. The improved conformity and more uniform contact pressure likely reduced surface mismatch and contributed to smoother adaptation of the seal faces.

Beyond 8 h, however, the torque began to increase gradually, followed by a series of pronounced peaks between 9 h and 14 h, with values exceeding 0.040 Nm. These peaks may be attributed to local instabilities such as adhesive events, uneven thermal expansion, or intermittent stick-slip phenomena resulting from the increased contact pressure. A sharp drop in torque near 14 h suggests temporary surface realignment, reduction in effective contact area, or wear-induced transitions in the interface.

Following this fluctuation, the torque stabilized at a higher level (~ 0.030 Nm) compared to SP0, although it continued to show low-frequency oscillations and a slow upward drift toward the end of the test. These behaviors likely reflect ongoing surface wear or evolving roughness under sustained loading. Importantly, the increase in spring preload resulted in a measurable rise in frictional torque, demonstrating a direct correlation between preload and interfacial shear forces. While improved preload enhances sealing reliability, it also intensifies frictional energy dissipation and may accelerate wear mechanisms over time.

### SP2—double contact pressure

Figure 3 presents the torque evolution for the SP2 configuration, where the contact pressure was doubled relative to SP1 configuration. Including the system baseline offset, this results in a total effective contact pressure of 0.826 MPa (Appendix Section B.2. Table 1) This setting produced a consistently elevated torque signal from the early stages of the test, with the average torque stabilizing near 0.070 Nm after 4 h. Although the early phase included minor transient peaks likely due to asperity interaction and initial plastic deformation, the system quickly converged to a high but stable torque plateau.

Compared to SP1, the SP2 configuration exhibited fewer and less pronounced dynamic fluctuations, suggesting that the excessive preload improved contact uniformity and reduced frictional instabilities. However, the sustained high torque levels imply a significant increase in interfacial shear forces and contact pressure. In dry-running conditions, this can lead to accelerated wear, thermal buildup, and possible degradation of the sealing surfaces.

A comparison across the three configurations reveals the clear influence of contact pressure on frictional torque behavior:

- SP0 (baseline contact pressure) resulted in the lowest torque levels (~ 0.015–0.020 Nm), with good long-term stability following a longer run-in phase.
- SP1 (recommended contact pressure) produced moderate torque values (~ 0.020–0.040 Nm) but was marked by distinct dynamic peaks and surface instabilities.
- SP2 (double contact pressure) yielded the highest and most stable torque (~ 0.065–0.070 Nm), though at the cost of increased friction and potential long-term material degradation.

These findings demonstrate that increasing the contact pressure not only raises the normal force between the seal faces but also fundamentally alters the nature of interfacial contact. The system shifts from a lightly loaded regime characterized by surface adaptation (SP0), through a transitional state with heightened dynamics (SP1), to a fully conformal, high-friction regime under SP2 conditions. While higher contact pressure improves contact reliability and torque consistency, it also increases wear risk and thermal loading. Therefore, contact pressure must be carefully optimized to balance initial sealing effectiveness with long-term tribological performance, particularly in dry-running applications where lubrication cannot mitigate contact stresses.

**Fig. 4:**
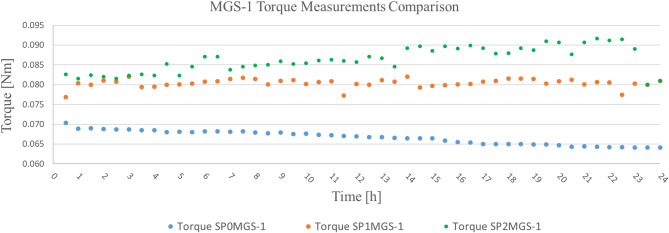
Measured torque over the 24-hour test duration for the SP0MGS-1, SP1MGS-1, SP2MGS-1 configuration. Average margin of errors are 0.61% for SP0MGS-1, 0.82% for SP1MGS-1, and 0.65% for SP2MGS-1.

The following diagrams (Fig. 4) summarize the results of the online measurements for SP0MGS-1, SP1MGS-1 and SP2MGS-1 measurements.

To evaluate the influence of regolith simulant contamination on the tribological performance of dry-running mechanical face seals, MGS-1 simulant (particle size PR < 80 μm) was introduced into the seal housing. The simulant was preloaded into the surrounding containment chamber prior to each experiment, but was not initially present in the sealing gap. Instead, it entered the interface during operation, mimicking realistic ingress scenarios encountered in extraterrestrial environments. Figure 4 shows the time-dependent evolution of frictional torque for the SP0MGS-1, SP1MGS-1, and SP2MGS-1 configurations, respectively.

Figure 4 presents the torque profile for the SP0MGS-1 case, where baseline contact pressure was applied. The initial torque was approximately 0.070 Nm, followed by a continuous, gradual decrease throughout the test duration. By the end of the experiment, the torque stabilized near 0.064 Nm. This monotonic decline suggests a mild polishing effect, likely caused by the continuous interaction between the sealing surfaces and the entrained simulant particles. The stable and smooth trend indicates that under minimal contact pressure, the interface remains resilient against frictional spikes, albeit at the cost of potentially reduced sealing force.

SP1MGS-1 corresponds to the nominal contact pressure condition. The torque rose quickly during the initial stage and remained relatively stable around 0.082 Nm. Although the profile included some low-frequency fluctuations and periodic sharp drops particularly in the latter half of the experiment no high-amplitude peaks were observed. These transients may be attributed to intermittent changes in the contact interface, such as localized particle buildup or ejection events. Relative to the clean SP1 case (steady-state torque ~ 0.030 Nm), this configuration showed a significant increase in friction, though the overall system remained dynamically stable.

The measured torque for SP2MGS-1 configuration began at around 0.085 Nm and gradually increased, exhibiting multiple fluctuations and pronounced peaks exceeding 0.095 Nm. A sudden drop occurred near the end of the measurement, settling at approximately 0.080 Nm. The elevated and unstable torque response implies intensified abrasive interactions between the simulant and the sealing interface under high contact pressure. Compared to the clean SP2 test, which showed a stable torque near 0.070 Nm, the presence of simulant introduced substantial dynamic variations and greater energy dissipation.

These measurements confirm that regolith simulant ingress significantly increases frictional torque in dry-running mechanical seals, with the severity of the response closely linked to the applied contact pressure. In particular:

- SP0MGS-1 showed the lowest and most gradually decreasing torque (~ 0.070 → 0.064 Nm), suggesting limited interfacial disturbance under low contact force.
- SP1MGS-1 maintained a relatively stable torque (~ 0.082 Nm), but exhibited transient dips, indicating partial disturbance mitigation.
- SP2MGS-1 produced the highest and most variable torque (peaks > 0.095 Nm), suggesting severe abrasive and thermomechanical effects.

The findings highlight the critical importance of contact pressure optimization in the presence of regolith simulant. While higher contact pressure enhances sealing pressure, it also increases the risk of frictional instability and wear due to intensified particle-surface interactions. Conversely, lower contact pressure configurations may promote smoother operation, but with potentially compromised sealing integrity. The SP1 condition offers a promising compromise, balancing operational stability with moderate torque levels even under simulant exposure.

**Fig. 5:**
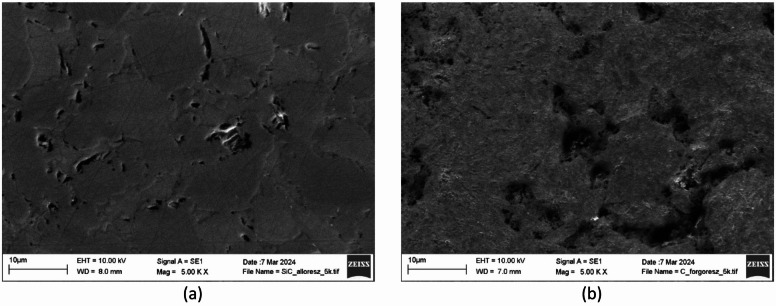
(**a**) SEM image of the SiC stationary face in as received condition, (**b**) SEM image of the graphite rotating face in as received condition.

The SEM image (Fig. 5a) represents the as-received condition of the silicon carbide seal face, prior to any exposure to MGS-1. The surface appears smooth with characteristic fine grinding marks and minimal surface defects, indicating a high-quality finishing process and clean baseline for tribological assessment.

Graphite is widely used as the rotating counterpart in mechanical seal assemblies, especially in conjunction with hard materials like silicon carbide or tungsten carbide. Its most critical advantage lies in its inherent self-lubricating property, which arises from its layered crystal structure. This allows for low friction operation even in dry or low-lubrication conditions. Furthermore, graphite exhibits good thermal resistance, chemical inertness, and the ability to conform slightly under pressure, enhancing sealing performance and reducing wear on the mating face.

As part of our tribological experiments involving MGS-1, this type of graphite rotating ring was used in contact with a silicon carbide stationary face. The SEM image (Fig. 5b) shown above depicts the graphite seal surface before exposure to the regolith, establishing a baseline for comparison. The scanning electron micrograph reveals the typical morphology of isostatically pressed graphite: a layered microstructure with visible pores and flake-like domains. These features contribute to graphite’s excellent dry-running behavior but may also serve as potential initiation sites for abrasive embedding when operating in dusty environments like Martian regolith. This initial surface condition allows for a clear before-and-after comparison with post-test SEM imaging, where wear mechanisms may be assessed.

**Fig. 6:**
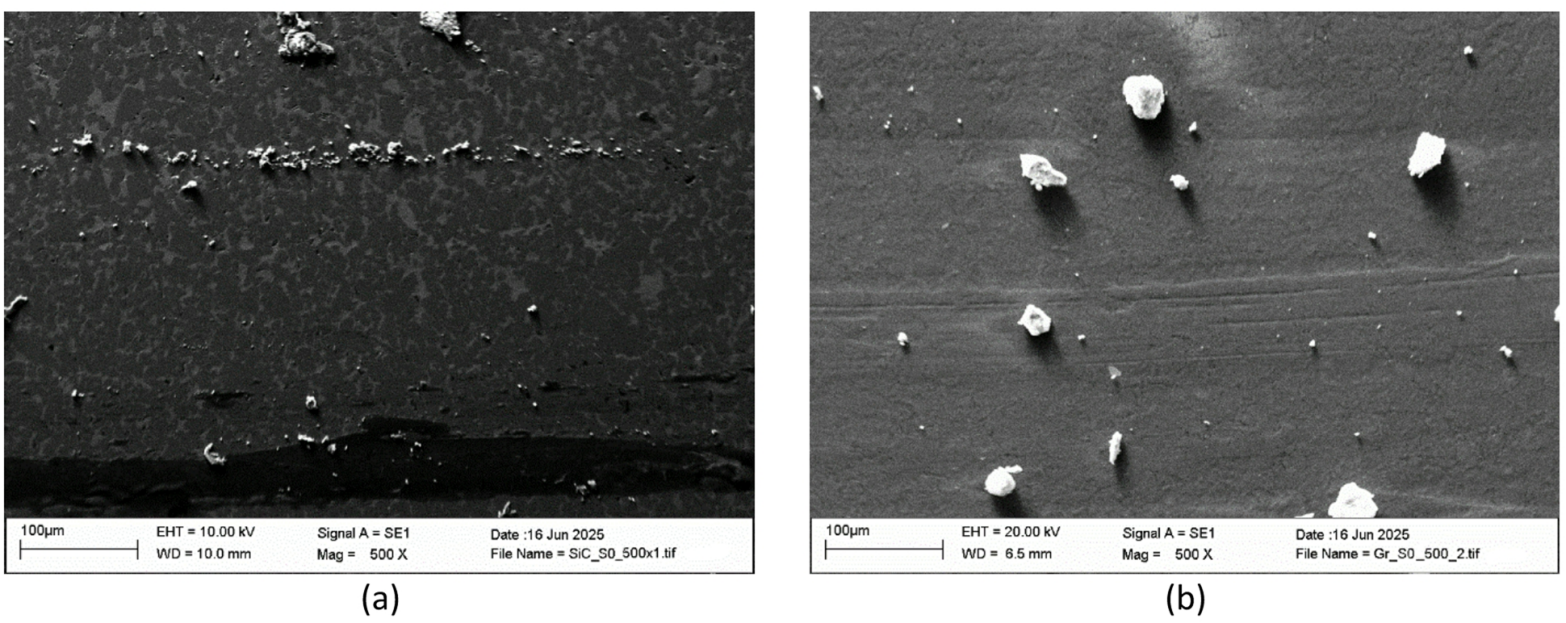
SEM record of the sealing surfaces after SP0 experiment with < 80 μm particle size MGS-1 (**a**) SiC, (**b**) graphite.

Following the completion of the reference test series (SP0, SP1 and SP2), the experimental program proceeded with the MGS-1 regolith simulant measurements, conducted sequentially in SP0, SP1, and SP2 configurations. To ensure a detailed characterization of the wear progression, the sealing components were disassembled from the test rig after each individual configuration for comprehensive documentation. Surface analysis was performed at each stage using scanning electron microscopy to track the evolution of surface degradation. The resulting surface conditions after SP0MGS-1 configuration are shown on Fig. 6a (SiC) and b (graphite).

Figure 6a shows the SEM micrograph of the silicon carbide surface after the SP0MGS-1 test phase. At this “technological zero” stage, the surface remains largely free of abrasive scratches or wear tracks. Instead, the primary observation is the random distribution of individual MGS-1 regolith particles adhering to the pristine SiC morphology.

Figure 6b illustrates the graphite sealing surface following the SP0MGS-1 test phase. In contrast to the SiC surface, the graphite exhibits noticeable surface deformation. The SEM micrograph reveals shallow, smooth track formations (smearing). Due to the inherent lubricity and lower hardness of graphite, the particles tend to press into the matrix or cause localized plastic deformation rather than sharp micro-cutting at this initial stage.

**Fig. 7:**
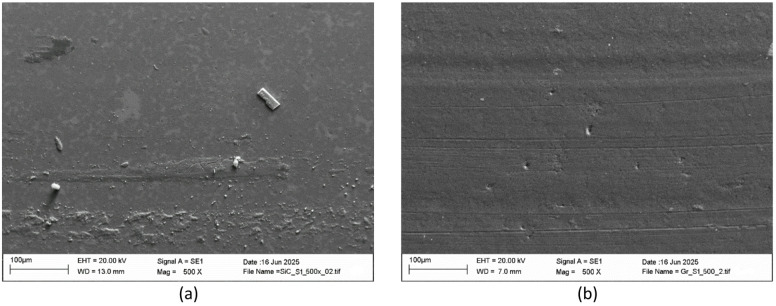
SEM record of the sealing surfaces after SP1 experiment with < 80 μm particle size MGS-1 (**a**) SiC, (**b**) graphite.

The silicon carbide surface after the SP1MGS-1 configuration remains remarkably similar to its appearance in the SP0MGS-1 phase. As shown in Fig. 7a, despite the transition from zero to nominal contact pressure, the surface morphology shows no significant changes or signs of abrasive wear. Following the cleaning and re-installation of the rings, the SEM micrograph reveals that the SiC surface remains free of scratches or scoring.

The graphite seal surface after the SP1MGS-1 test phase is shown in Fig. 7b. Utilizing the same ring from the previous SP0MGS-1 stage, the surface now exhibits a clear progression in wear intensity under nominal contact pressure. The SEM micrograph reveals prominent, continuous wear tracks and deeper smearing across the contact zone. The softer graphite matrix allows the MGS-1 particles to actively plow the surface, leading to visible plastic deformation and the formation of parallel grooves. This confirms that the graphite surface undergoes significant morphological transition as the contact pressure increases.

**Fig. 8:**
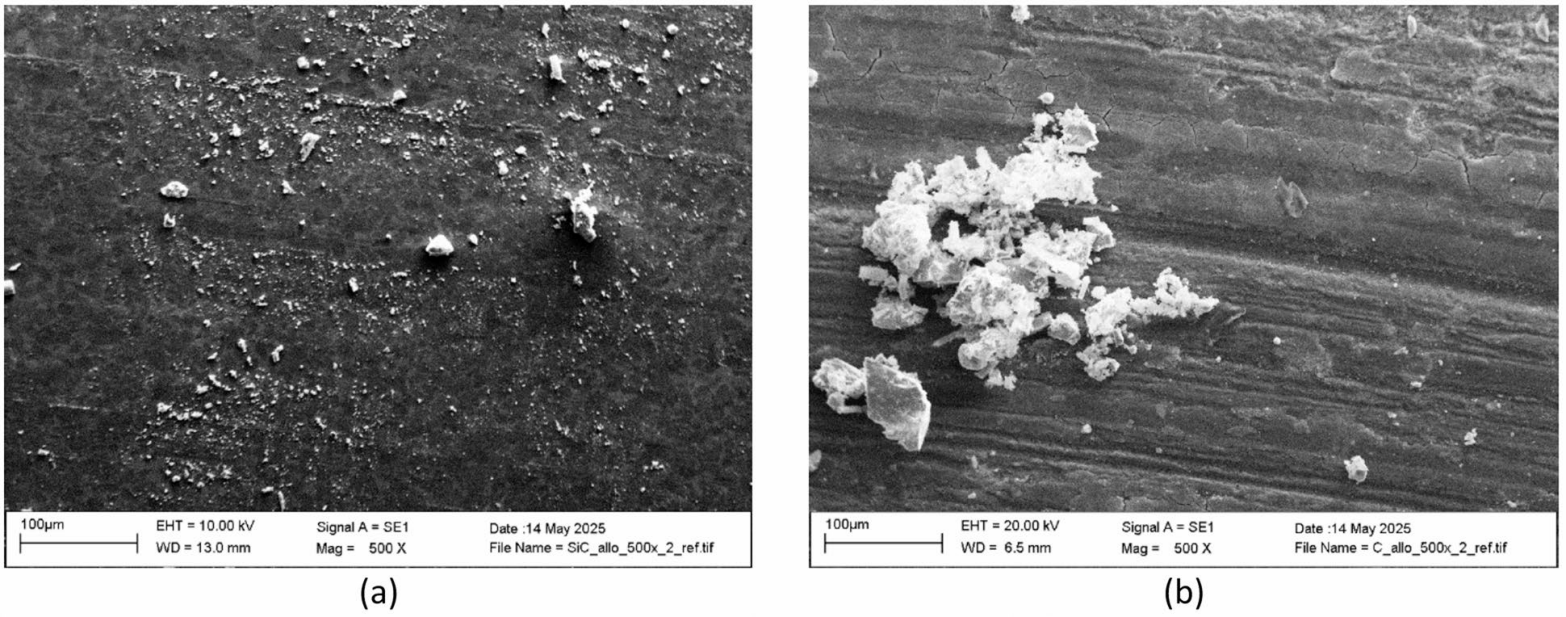
SEM record of the sealing surfaces after SP2 experiment with < 80 μm particle size MGS-1 (**a**) SiC, (**b**) graphite.

Figure 8a illustrates the SEM images of the silicon carbide (SiC) stationary seal face displayed an almost intact, slightly polished surface. No traces of scratches or grooves can be developed on the surface, despite the presence of fine particles of the Martian regolith, primarily sub-micrometer in size, adhered to it. In light of the significant difference in the relative hardness of the regolith containing mineral phases and that of the SiC, this outcome was to be expected.

The SEM analysis of the graphite rotating seal face revealed surface features consistent with abrasive wear due to fine regolith particle interaction. Unlike the SiC surface, the graphite material exhibited a heterogeneous wear pattern, likely influenced by its softer, layered microstructure and natural porosity. The image showed (Fig. 8b) widespread surface deformation, with numerous shallow grooves and worn zones aligned predominantly along the direction of rotation. These features suggest continuous sliding contact and mechanical erosion during the test.

The experiment was completed under controlled conditions using a sealed housing filled with fine MGS-1 regolith simulant (particle size below 80 μm) to simulate a dust-laden Martian environment. The mechanical seal assembly, consisting of a silicon carbide (SiC) stationary face and a graphite rotating face, was mounted under standard loading conditions. An electrical drive system induced rotation of the shaft, creating relative motion between the two seal faces. During the test, no human-induced contamination occurred; the fine MGS-1 regolith naturally entered the seal face interface through operation.

In several regions, the graphite surface displayed localized compaction and smearing, possibly caused by the pressure and heat generated at the interface. These phenomena are typical for carbon-based materials under frictional load, where the uppermost layers may deform plastically or fracture along their grain boundaries. In addition, small particles resembling trapped or partially embedded regolith grains were visible on the surface, further confirming the abrasive action of the MGS-1 simulant.

### Post-test tribological analysis and SEM imaging

Following the 24-hour test runs, the mechanical seal faces were disassembled and examined using Scanning Electron Microscopy (SEM) to identify the wear mechanisms induced by the MGS-1 regolith simulant. This analysis provides visual evidence that directly supports the frictional torque data presented in the previous section.

On the tested silicon carbide (SiC) stationary face the surface remains largely smooth and free of abrasive scratches, maintaining its structural integrity even under double nominal pressure. Close inspection reveals that while no grooves or pitting developed, there is a notable presence of adhering MGS-1 particles. Unlike in softer materials, these particles are not embedded into the SiC matrix due to the ceramic’s extreme hardness; instead, they remain securely adhered to the surface. These adhering particles can act as localized asperities, potentially contributing to the recorded friction torque. The high hardness of SiC effectively prevented surface damage, proving that the material’s resistance is a critical factor in the seal’s performance under regolith exposure.

The tested graphite rotating face, in contrast, exhibits more severe damage. The surface is covered with deep, parallel grooves, a classic sign of three-body abrasive wear. This occurred as the fine regolith particles became trapped between the rotating graphite and the stationary SiC, causing significant material removal. Furthermore, large agglomerations of wear debris a mixture of graphite particles and MGS-1 simulant are visible on the surface. This accumulation of consolidated material likely contributed to the dynamic fluctuations and spikes observed in the torque data for the simulant-contaminated tests.

These post-test SEM images provide direct, visual evidence of the tribological mechanisms at play. They confirm that the MGS-1 simulant acts as a highly abrasive medium, causing significant surface damage that explains the increase in frictional torque and compromises the long-term integrity of the seal, particularly in the softer graphite component. Some areas also showed micro-fractures and edge chipping, indicating that brittle failure may have occurred under localized high-stress conditions. This type of damage is especially relevant for evaluating the long-term performance of the seal in Martian regolith environments, where sharp particle edges and cyclic loading could exacerbate fatigue.

### Analysis of frictional behavior and particle-surface interactions

The Coefficient of Friction (COF) for the mechanical seal was calculated using the following formula for the dry-running conditions:

$$\:\mu\:=\frac{T}{{(F}_{axial}+{F}_{base})\cdot ravg}$$

Where:

- µ is the Coefficient of Friction (COF).
- T is the measured frictional torque from the seal interface, in Nm.
- F_axial_ is the applied axial force from the spring preload, in N.
- F_base_ is the equivalent baseline force (systemic preload) [N].
- r_avg_ is the average radius of the sealing contact surface, in m.

Using the determined inner diameter of 16.25 mm and the outer diameter of 18.35 mm, the average radius was calculated to be 8.65 mm. Based on this and the measured torque and axial force values, the calculated COF for each configuration is presented in Table 1. The calculated baseline force of 13.1 N (corresponding to 0.23 MPa) is considered a systemic preload. This value represents the combined effect of the inherent friction of the bearing assembly and the initial contact established during the assembly process.

**Table 1:** Calculated COF for each configuration.

Configuration	Total effective force (F_eff_) [*N*]	Frictional torque (T) [Nm]	Calculated COF (µ)
SP0 (clean)	13.1	0.017 ± 0.002	0.150^*^
SP1 (clean)	30.1	0.030 ± 0.003	0.115 ± 0.012
SP2 (clean)	47.1	0.070 ± 0.005	0.172 ± 0.014
SP0MGS-1	13.1	0.065 ± 0.006	0.573 ± 0.053
SP1MGS-1	30.1	0.082 ± 0.007	0.315 ± 0.028
SP2MGS-1	47.1	0.095 ± 0.008	0.233 ± 0.021

^*^Reference value sourced from literature^22^.

The residual torque (0.017 Nm) measured in this state results from the followings:

- System characteristics: During assembly, the seal faces are brought into contact to close the sealing gap, but no active spring preload was applied during operation. The observed torque is a characteristic of the assembly itself, resulting from the inherent friction of the bearing assembly, elastic resistance and self-weight of the components.
- Adhesive component: Even without external mechanical loading, the finely ground graphite and SiC surfaces experience adhesive attraction. This adhesive component of friction is non-zero, as the molecular forces between the mating surfaces maintain a baseline contact, generating a measurable resistance torque even in the absence of spring-induced normal force.

To provide a physically consistent friction model, this residual torque was converted into an equivalent baseline force (F_base_ = 13.1 N), corresponding to a baseline contact pressure of 0.23 MPa. By incorporating this offset into the Amontons-Coulomb model, a representative COF for the SP0 state was determined (µ ≈ 0.15).

The data reveals a key counterintuitive finding: the Coefficient of Friction (COF) for the SP2MGS-1 configuration (0.233) is significantly lower than that of the SP1MGS-1 configuration (0.315), despite the higher normal load. This phenomenon highlights a critical aspect of tribological behavior in particulate-conta minated environments.

The higher normal force in the SP2 configuration leads to increased contact pressure at the seal interface. This intense pressure facilitates the embedding of abrasive regolith particles into the softer graphite surface, effectively removing them from the tribologically active contact zone. By burying the “third-body” particles, the system transitions from an aggressive three-body abrasive regime to a more stable contact scenario, where the remaining friction is dominated by the interaction between the now-modified surfaces. Furthermore, the higher pressure may also promote the formation of a stable, compacted third-body layer composed of graphite wear debris and simulant particles. This consolidated layer can act as a solid lubricant, reducing the direct surface-to-surface contact and thereby lowering the overall friction.

Conversely, in the SP1MGS-1 configuration, the lower contact pressure is insufficient to embed or consolidate the regolith particles, allowing them to freely roll and slide between the surfaces. This dynamic third-body action leads to more aggressive abrasion, higher energy dissipation, and a substantially elevated COF. This observation confirms that in dry, dusty environments, a higher normal load does not necessarily lead to proportionally higher friction; instead, it can fundamentally alter the wear mechanism, potentially improving operational stability.

## Discussion

The tribological experiments conducted under both clean and simulant-contaminated conditions provide key insights into the wear mechanisms and frictional behavior of dry-running mechanical seals composed of a silicon carbide (SiC) stationary face and a graphite rotating ring. The variation in torque behavior across different contact pressure configurations, combined with the scanning electron microscopy (SEM) observations, allows for a detailed evaluation of the seal system’s response to mechanical loading and particulate ingress.

### Effect of contact pressure on frictional behavior

Under clean conditions (SP0, SP1, SP2), the torque data demonstrated a clear relationship between contact pressure and interfacial shear forces. Low contact pressure (SP0) resulted in minimal and stable torque after an extended run-in period, highlighting the capacity of graphite–SiC pairs to operate efficiently under gentle contact pressure. The absence of dynamic frictional spikes in this configuration suggests that such low-pressure conditions promote self-lubricating behavior with minimal interfacial instability.

In contrast, the single contact pressure (SP1) and the doubled contact pressure (SP2) led to an increase in average torque and introduced characteristic torque fluctuations. These instabilities are primarily attributed to the adhesive wear mechanisms and the stochastic nature of the graphite transfer film formation on the SiC counterface. Synchronous monitoring of the axial force during these phases indicated minor fluctuations (± 2–3%) that correlate directly with the torque peaks. This behavior suggests a “stick-slip” like interaction at the microscopic level: as the graphite asperities interact with the harder SiC surface, localized adhesive bonds are formed and subsequently sheared. The periodic spikes in torque represent the moments of increased adhesive resistance before the local shear failure of the graphite transfer layer. Furthermore, the axial force data suggests that microscopic changes in the contact topography, such as the generation and temporary entrapment of fine graphite wear debris cause transient variations in the actual contact pressure, leading to the observed local instabilities. This highlights the inherent trade-off between contact reliability and wear acceleration in contact pressure-dependent dry-running systems, where higher contact pressures improve conformity but increase the complexity of the interfacial adhesive dynamics.

### Impact of simulant ingress

In the presence of MGS-1 regolith simulant (Figs. 4), the friction torque characteristics underwent a fundamental shift compared to clean operation. All configurations exhibited significantly elevated torque levels, confirming the severe abrasive impact of the particulate matter. These phenomena are characteristic of third-body abrasion, as defined by Berthier^27^, where the regolith particles act as an interfacial medium, the “third body” that governs the contact mechanics.

The observed wear mechanisms on the sealing surfaces, documented by SEM, clearly indicate the transition from adhesive to abrasive dominance. The hard basaltic particles (500–1000 HV) of the MGS-1 simulant, being significantly harder than the graphite ring (80–100 HV), caused severe surface degradation. The primary micro-mechanisms identified include plastic deformation of the graphite matrix and wedge formation, where particles become partially embedded and plow through the surface. The high degree of penetration of the angular simulant grains led to extensive micro-cutting, which is evidenced by the deep, continuous grooves visible in the post-test surface topography.

The sudden drops and instabilities observed in the torque measurements (Fig. 4), especially in the SP1 and SP2 cases, are attributed to the dynamic behavior of the regolith particles within the sealing gap. These particles are not stationary; they continuously rearrange, fragment, or get expelled from the interface. The transient torque drops represent moments when the “third body” layer temporarily destabilizes or when a cluster of particles is crushed and redistributed, momentarily reducing the interfacial resistance before new grains enter the contact zone.

In the SP0 condition, a monotonic decrease in torque was observed, suggesting that under minimal contact pressure, the abrasive particles primarily perform a polishing action with a lower degree of penetration, gradually stabilizing the interface. In contrast, under higher contact pressures (SP1 and SP2), the particles are pressed into the graphite with greater force, leading to the aforementioned micro-cutting and stochastic torque fluctuations. This highlights the dominant role of the third-body flow effects, as described by Berthier, in explaining the non-linear and unstable friction behavior observed under Martian simulant exposure.

### SEM-based wear characterization

Post SEM imaging corroborated the recorded torque data and provided direct evidence of the dominant wear mechanisms. The graphite rotating ring exhibited extensive surface degradation, consistent with its inherently lower hardness (approx. 80–100 HV) relative to the MGS-1 regolith. The observed topography is characterized by deep groove formations and clear signs of micro-cutting, where the angular basaltic particles achieved a high degree of penetration into the layered graphite structure. Localized plastic deformation, wedge formation, and smearing of graphite debris were also prominent, particularly under SP1 and SP2 conditions. These features confirm the establishment of a complex third-body layer, as theorized by Berthier^27^, where the trapped simulant particles act as abrasive agents that continuously reshape the contact interface.

In contrast, the SiC stationary face (approx. 2200 HV) largely retained its initially smooth, finely ground characteristic due to its superior hardness. However, SEM analysis revealed residual simulant particles and transferred graphite fragments adhered to the surface. This suggests that while the SiC resists direct abrasive cutting, it acts as a substrate for the stochastic distribution of the third-body phase, where particles can become temporarily trapped or crushed, leading to the transient torque fluctuations and “stick-slip” events observed during the experiments.

While the torque increase under clean conditions follows the classical Amontons–Coulomb laws^28^, the introduction of regolith simulant shifts the focus to abrasive degradation mechanisms. The observed groove formations and surface damage are not merely consequences of increased friction, but represent a fundamental change in the micro mechanisms of wear. According to the models proposed by Rabinowicz^29^ and Hutchings^30^, the high degree of penetration of the angular MGS-1 particles leads to severe micro-cutting, where the volume of material removed is governed by the abrasive phase’s geometry and hardness rather than simple interfacial sliding. Consequently, the central finding of this study is not the torque-load proportionality itself, but the identification of the threshold where preload accelerates the transition from mild surface polishing to catastrophic abrasive failure.

Furthermore, while the increase in wear with applied load can be broadly described by the Archard wear model^31^, which establishes a linear relationship between wear volume, normal load, and sliding distance, our results indicate localized deviations due to the presence of the abrasive third body. Under regolith contamination, the wear is not merely a function of a global Archard coefficient (*k*), but is governed by the dynamic entrapment, fragmentation, and “flow” of particles as described by Berthier^27^. The SEM observations confirm that the severity of the micro-cutting process exceeds the predictions of simple adhesive wear models, emphasizing that in planetary environments, the “abrasive potential” of the regolith overrides the classical Archard-type steady-state assumptions.

### Practical implications for extraterrestrial applications

The findings of this study have direct relevance for the design of mechanical face seals for future planetary exploration missions, where exposure to fine, abrasive regolith is a critical failure mode. Our results demonstrate that the friction and wear behavior is governed by the dynamic equilibrium of the third-body layer (regolith and graphite debris) within the sealing interface.

While increasing the spring preload enhances the contact pressure and theoretically improves the exclusion of external contaminants, it also promotes a higher degree of penetration for the trapped abrasive particles. This leads to accelerated micro-cutting and increased frictional energy dissipation, as seen in the SP2 configurations. Conversely, a very low contact pressure (SP0) may fail to maintain a stable sealing interface, allowing for erratic particle ingress and potential leakage.

The SP1 condition emerged as the most balanced solution, providing sufficient contact pressure to stabilize the graphite transfer film while maintaining the torque fluctuations within a manageable range. This suggest that for long-term deployment in dusty extraterrestrial environments, such as the Martian surface, seal design must optimize the preload to facilitate “third-body” stability. A carefully calibrated preload optimized to balance sealing integrity against the stochastic abrasive risks of regolith is essential to maximize the operational lifespan and energy efficiency of rotating mechanisms in space exploration.

## Conclusion

This study provides an initial tribological approximation of dry-running mechanical face seals, which are critical components for protecting electromechanical systems in demanding extraterrestrial environments, such as Mars. While the current investigation focused on the mechanical and particulate-related aspects of seal performance under ambient conditions, it serves as a foundational step toward understanding the complex interactions at the seal interface. By testing a graphite–silicon carbide seal pair under varying spring preload conditions and in the presence of MGS-1 regolith simulant, our findings provide crucial insights into the interplay between mechanical loading, particle ingress, and tribological durability.

Our analysis confirms a clear trade-off between sealing integrity and tribological wear. Under clean conditions, the friction torque was primarily governed by adhesive mechanisms and the formation of a graphite transfer film, where synchronous monitoring of the axial force revealed that torque fluctuations correlate with microscopic “stick-slip” events. The introduction of fine MGS-1 regolith simulant fundamentally shifted the wear regime to third-body abrasion, as theorized by Berthier. The simulant significantly elevated torque levels across all configurations, acting as an aggressive abrasive medium.

Post-test SEM analysis visually corroborated these findings, showing distinct abrasive wear on the graphite face. The primary micro-mechanisms identified were plastic deformation, wedge formation, and micro-cutting, characterized by deep grooves and a high degree of penetration of the basaltic particles into the softer graphite matrix (80–100 HV). In contrast, the much harder silicon carbide (2200 HV) remained practically intact, serving mainly as a substrate for the stochastic distribution and crushing of the third-body phase.

Most importantly, our data revealed that the SP1 configuration offers the most resilient balance between operational stability and long-term durability. While the highest contact pressure led to a higher overall torque and greater risk of material degradation through intensified micro-cutting, the SP1 setting maintained a more stable torque profile with moderate wear.

The experimental outcomes further reveal that increasing the contact pressure does not provide a protective effect against abrasive degradation from fine particulate ingress. Instead, elevated contact pressure accelerates the wear of the graphite ring and introduces an unnecessary energetic penalty. For practical applications in particulate-rich environments, these findings stress the importance of optimizing preload levels to stabilize the third-body flow rather than assuming that bigger contact pressure inherently improve seal protection. These findings may contribute to the design of future planetary probes and exploration systems, where energy efficiency and resistance to regolith contamination are of paramount importance.

## Supplementary Information

Supplementary Material 1

## Data Availability

The data that support the findings of this study are available from the corresponding author upon reasonable request.
