# Supplementary material for: Tribological performance of mechanical face seals for Martian applications
Source: Sci Rep. 2026 Apr 14;16:18459. doi: 10.1038/s41598-026-48495-2 (PMC13265907; doi:10.1038/s41598-026-48495-2)
Supplement: Supplementary file 1 — Supplementary Material 1 [file 41598_2026_48495_MOESM1_ESM.docx]

**Supplementary Material for:** Tribological performance of mechanical face seals for Martian applications

**Authors:** Marcell Péter Kiss, Gábor Kalácska, Róbert Zsolt Keresztes, Zoltán Károly

**Content:** This document provides technical details regarding the experimental test rig, spring calibration, and contact pressure calculations.

**Section A.1: Detailed Description of the Tribological Test Rig and Sensors**

The experiments were conducted using a custom-developed tribological test rig designed for stable rotational speeds and precise torque measurement.

- **Drive and Transmission:** The system is driven by a three-phase asynchronous electric motor, with its rotational speed precisely controlled by a variable frequency drive (VFD). At the end of the motor shaft, a belt transmission transfers torque to the main shaft.
- **Rotational Speed Monitoring:** The speed is continuously monitored by an optical sensor (Baumer DF10) mounted at the output of the belt drive.
- **Shaft Assembly and Support:** The torque is transmitted along a single, continuous shaft, supported by a bearing housing that ensures proper alignment while constraining radial and axial displacement. The shaft terminates in a blind flange containing a rolling-element bearing for additional radial support.
- **Torque Measurement System:** A torque transducer (HBM T22) is installed between two flexible couplings to allow direct and accurate measurement of net torque, decoupled from torsional vibrations.
- **Friction Torque Quantification:** The right side of the dust chamber is mounted onto a fixed bearing housing. This allows the chamber to rotate slightly under the influence of frictional forces. This reaction torque is quantified by a force transducer (MT1022-3KG-3M) connected via a rigid arm to the chamber body, measuring the tangential force from which dynamic friction torque is calculated.
- **Data Acquisition:** The system utilizes a data acquisition unit (Spider 8) connected to a computer to record all sensor outputs.

**Section A.2: Friction Torque Quantification and Baseline Measurements**

The sealing pair consists of a stationary Silicon Carbide (RBSiC) ring and a rotating resin-impregnated carbon-graphite ring (Calvo Sealing). The stationary ring is mounted in a specialized housing, while the rotating ring is fixed to the shaft. To ensure perfect alignment and zero initial clearance, the contact integrity was verified using a light-gap test before each test run. The precise geometry (see **Figure 1**) of the contact interface is as follows:

Outer Diameter (*d_out_*): 18.35 mm

Inner Diameter (*d_in_*): 16.25 mm

Nominal Contact Area (*A*): 57.07 mm²

$$A=\frac{\pi}{4}\cdot\left( d_{out}^{2}-d_{in}^{2} \right)=\frac{\pi}{4}\cdot\left( {18,35}^{2}-{16,25}^{2} \right)\approx57,07{mm}^{2}$$

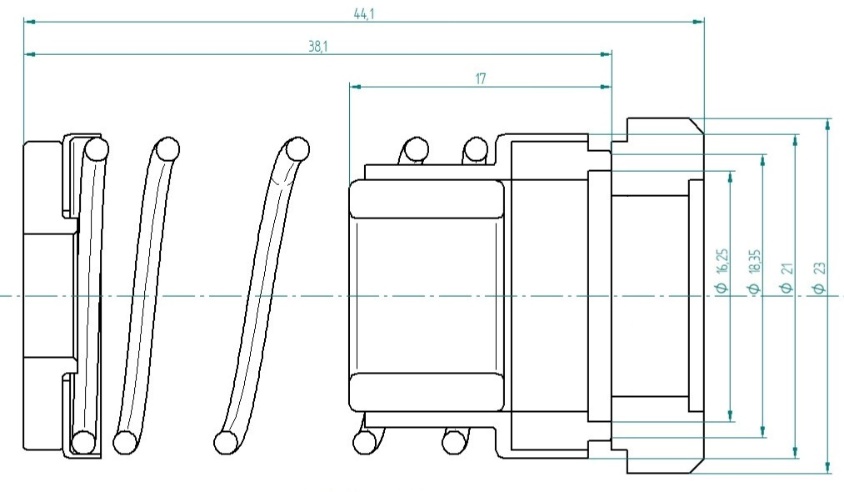


**Figure 1**. Cross-section layout of the mechanical seal component

**Dust Chamber and Simulant Delivery**

The sealing interface is enclosed within a horizontal annular dust chamber. To simulate the abrasive Martian environment, 150 ml of MGS-1 simulant (particle size < 80 µm) was introduced into the chamber via a top-fill port. (see **Figure 2**) In this horizontal arrangement, the simulant initially settles at the bottom of the chamber. Upon starting the rotation, ensured that the entire circumference of the sealing interface is continuously exposed to the abrasive regolith simulant, mimicking the ingress of dust in Mars rover components.

**Instrumentation and Data Acquisition**

Frictional torque was monitored using a high-sensitivity torque transducer integrated into the drive train. To manage the large data volume over the 24-hour testing period while ensuring a stable representation of the friction process, a specific data acquisition strategy was implemented. The experimental procedure consisted of 48 discrete measurement cycles, where each cycle comprised a 5-second active sampling window at a frequency of 10 Hz, followed by a 1795-second idle period. This approach yielded 50 raw data points per cycle.

To enhance the signal-to-noise ratio and minimize high-frequency drive-train vibrations in the final visualizations, the recorded values within each cycle were processed using a segmental block-averaging technique. Specifically, the data were decimated by averaging every 10 consecutive samples into a single representative value, resulting in five smoothed data points per measurement cycle. This strategy effectively suppressed transient stochastic noise while preserving the macroscopic frictional trends, providing 240 statistically robust data points to generate the time-dependent torque curves presented in this study.

**Measurement of Friction Torque**

The friction torque (*M_f_*) was determined indirectly using a reaction-force measurement principle. The dust chamber assembly is mounted on low-friction bearings, allowing it to rotate slightly under the influence of the seal interface friction. This rotation is constrained by a rigid radial lever arm (rod) that remains in constant contact with a high-precision MT1022 load cell. See **Figure 2.**


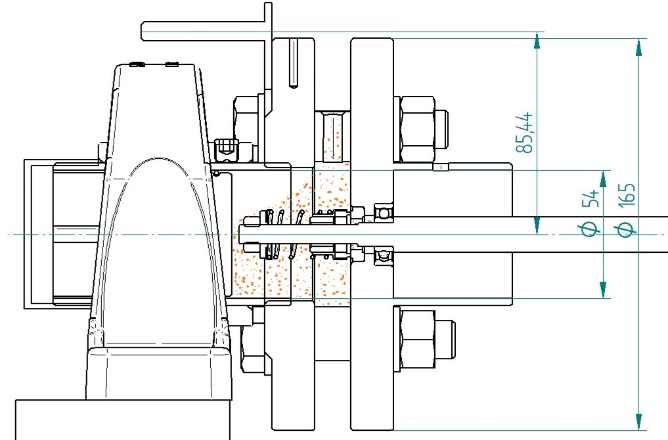


**Figure 2.** Cross-Sectional view of the mechanical seal test setup and simulant delivery into the chamber

The friction torque is calculated as the product of the measured reaction force and the length of the lever arm:

$$M_{f}=F_{c}\cdot L$$

Where:

- *F_c_* is the reaction force measured by the load cell [N].
- *L* is the length of the lever arm, defined as the distance between the center of the shaft and the contact point of the load cell, which is 85 mm in this setup.

This configuration ensures that only the friction generated at the sealing interface is measured, effectively isolating it from any parasitic friction originating from the motor, belt transmission, or drive shaft bearings.

**Section B.1: Spring Calibration Procedure and Stiffness Determination**

The axial preload (*F_n_*) applied to the mechanical seal interface was provided by a precision-ground compression spring. To ensure the reliability of the calculated contact pressure values, a dedicated calibration procedure was performed prior to the tribological testing.

**Calibration Procedure**

The force-displacement characteristic of the spring was determined using a high-precision calibration stand. The spring was positioned on a calibrated load cell mounted on a rigid base. The compressive load was applied axially by a micrometer-precision vertical plunger. The displacement (*Δl*) of the plunger was monitored using an integrated digital linear scale with a resolution of 0.01 mm, while the corresponding reaction force (*F_n_*) was recorded by the load cell. Measurements were taken in 1 mm increments across the entire operational range.

**Section B.2: Contact Pressure Calculations**

The load – deflection characteristics of the preload ring is shown on **Figure 3**. The spring constant (*k*) was determined from the slope of the linear regression fitted to the experimental data:

$$F_{n}=k\cdot\Delta l$$

The spring constant for the specific component used in the test rig was found to be *k* = 1.975 N/mm.


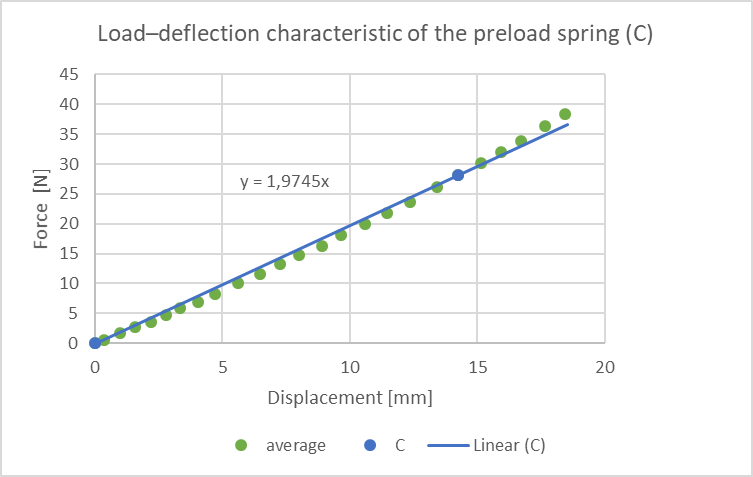


**Figure 3**. Measured spring force–displacement characteristic (C) with linear regression (k ≈ 2 N/mm).

**Preload Setting**

In the tribological experiments, the spring compression was set by adjusting a self-locking nut at the end of the shaft. The displacement (*Δl*) was verified using a digital caliper with a resolution of ±0.01 mm. The two tested conditions were established as follows:

SP1 (Nominal preload): *Δl* = 8.60 mm →*F_n_* = 17.0 N

SP2 (Double preload): *Δl* = 17.20 mm →*F_n_* = 34.0 N

The average contact pressure resulting from spring preload (*p_avg_*) was calculated as the ratio of the axial force to the nominal contact area of the seal faces (*A* = 57.07 mm^2^) in **Table 1**.

$$P_{avg\_sp1}=\frac{F_{n}}{A}=\frac{17}{57.07}=0.298 MPa$$

$$P_{avg\_sp2}=\frac{F_{n}}{A}=\frac{34}{57.07}=0.596MPa$$

To account for the mechanical baseline of the assembly, a system baseline offset P_base_ = 0.230 MPa was estimated from the residual torque at zero external load (SP0). The total effective pressure for each configuration is summarized in **Table 1**.

**Table 1.** Mechanical parameters and effective contact pressures of the tested configurations.

| **Configuration** | **Spring compression (mm)** | **Spring force (N)** | **System Baseline Offset (MPa)** | **Average Contact Pressure from Spring Preload (MPa)** | **Total Effective pressure (MPa)** |
| --- | --- | --- | --- | --- | --- |
| SP0 (baseline) | 0.0 | 0.0 | 0.230 | 0.000 | 0.230 |
| SP1 (single) | 8.6 | 17.0 | 0.230 | 0.298 | 0.528 |
| SP2 (double) | 17.2 | 34.0 | 0.230 | 0.596 | 0.826 |

**Section B.3: Uncertainty Analysis**

**Torque measurement:** The torque transducer has a calibrated accuracy of ± 0.5% of the full scale.

**Force application:** Based on the spring constant (*k* = 1.975 N/mm) and the digital caliper resolution (±0.01 mm), the uncertainty in the axial force application is approximately ±0.02 N.

**Pressure precision:** This leads to a contact pressure uncertainty of less than ±0.001 MPa, which confirms the stability of the SP1 and SP2 conditions.

**Load Cell:** The load cell has a combined error ≤0.016 %.

**Section C.1: Material Specifications**

To interpret the wear mechanisms observed on the graphite rotating ring, the physical and mechanical properties of the MGS-1 (Mars Global Simulant) were considered in relation to the seal face materials.

**Properties of MGS-1 regolith**

The MGS-1 was selected for this study due to its high fidelity in replicating the mineralogical and chemical composition of Martian soil. The simulant is primarily composed of crushed basaltic rock and selected mineral additives to match the crystalline-to-amorphous ratio found at Gale Crater. Significant characteristics of the selected regolith is listed in **Table 2**.

**Table 2.** Significant characteristics of the MGS-1 regolith simulant.

| **Regolith Simulant** | **MGS-1** |
| --- | --- |
| **Original Material** | Average Martian Soil |
| **Phase Composition** | Anorthosite 27.1% |
|  | Glass-rich basalt 22.9% |
|  | Pyroxene 20.3% |
|  | Olivine 13.7% |
|  | Mg-sulfate 4.0% |
|  | Ferrihydrite 3.5% |
|  | Hydrated silica 3.0% |
|  | Magnetite 1.9% |
|  | Anhydrite 1.7% |
|  | Fe-carbonate 1.4% |
| **Physical Properties** | Bulk density: 1.29 g/cm³ |
|  | Median particle size: 60 µm |
|  | Particle size range: >0.04–600 µm |
| **Reference** | [22] |

**Material Hardness Comparison**

The MGS-1 simulant is a high-fidelity basaltic regolith proxy. Its mineralogical composition (primarily plagioclase, pyroxene, and olivine) results in a high abrasive potential. The hardness contrast between the particles and the seal faces is summarized below:

- **Stationary Ring (RBSiC):** ~2200 HV (see **Table 3**), which is significantly harder than the simulant particles, providing excellent resistance against abrasive wear.
- **MGS-1 Simulant:** The constituent minerals possess a Mohs hardness of 6–7, which corresponds to approximately 700–900 HV. [17]
- **Rotating Ring (Resin-impregnated Graphite):** The Shore hardness is approximately 80–90 HSD, which is orders of magnitude lower than that of the simulant particles.

**Table 3.** Reaction Bonded Silicon Carbide Technical Data

| **Operating Limits** | **Units** | **SiC** |
| --- | --- | --- |
| **Volume density** | g/cm3 | ≥ 3.03 |
| **Hardness** | HV_0.5_ | ≥ 2200 |
| **Indicated Porosity** | % | < 0.2 |
| **Compressive Strength** | MPa | ≥ 2000 |
| **Flexural Strength** | MPa | ≥ 350 |
| **Free-si value** | % | < 12 |
| **Purity (SiC Percentage)** | % | ≥ 98 |
| **Elastic Modulus** | GPa | 350 |
| **Thermal Conductivity** | W/m · K | 50~100 |
| **Maximum Temperature** | °C | 1000 |
| **Coefficient of Heat Expansion** | 10-6 ·1/°C | 4.0 |

**Section D.1: Theoretical Framework of the Three-Body Abrasive Wear Mechanism**

The observed scratches and material loss on the graphite surface are a direct consequence of this hardness gradient. In the presence of the simulant, the process follows a three-body abrasive wear model:

- **Particle Entrapment:** Due to the relatively low hardness and porous nature of the graphite, the fine MGS-1 particles (*<80* µm) can become temporarily embedded or "trapped" in the softer graphite surface.
- **Micro-cutting:** As the shaft rotates, these trapped or free-rolling hard basaltic particles act as micro-cutters against the graphite face.
- **Preferential Wear:** Since the Silicon Carbide (RBSiC) ring remains largely unaffected due to its superior hardness (*≥2200* *HV*), the abrasive energy is almost entirely dissipated through the wear and grooving of the sacrificial graphite ring.

**Particle Size Distribution**

The simulant used in this study was sieved to a fraction of *<80* µm. This size range is particularly critical for mechanical face seals, as the particles are small enough to approach the nominal sealing gap but large enough to cause significant micro-ploughing once they enter the interface under the influence of centrifugal and axial forces.
